# Stem design affects templating adherence in total hip arthroplasty - a retrospective cohort study comparing two types of cementless short stems.

**DOI:** 10.1186/s13018-025-05801-4

**Published:** 2025-04-17

**Authors:** C. Stadler, A. Edinger, B. Schauer, DJ. Haslhofer, T. Gotterbarm, M. Luger

**Affiliations:** 1 Johannes Kepler University Linz, Altenberger Strasse 96, Linz, 4040 Austria; 2 Department for Orthopaedics and Traumatology, Kepler University Hospital GmbH, Med Campus III, Krankenhausstraße 9, Linz, 4020 Austria

**Keywords:** Total hip arthroplasty, Templating, Cementless short stem, Direct anterior approach, Neck-resecting, Neck-sparing

## Abstract

**Background:**

Preoperative templating is crucial when performing total hip arthroplasty (THA) as it facilitates the correct restoration of the joint biomechanics and reduces the risk of adverse events associated with component under- or over-sizing. Templating and execution of stem placement is highly dependent on the actual stem design. Therefore, we aimed to compare the templating adherence between a neck-resecting and a partially neck-sparing cementless short stem and to evaluate the influence of patient-specific factors like sex and Dorr type on the templating adherence.

**Methods:**

This retrospective cohort study evaluated the preoperative templates of 345 consecutive THAs performed by a single surgeon. A neck-resecting short stem (Fitmore, ZimmerBiomet) combined with a bi-hemispherical cup (Allofit, ZimmerBiomet; Group A) was used in 160 cases and a partially neck-sparing short stem (ANA NOVA alpha proxy, ImplanTec GmbH) combined with a bi-hemispherical cup (ANA NOVA alpha cup, ImplanTec GmbH; Group B) in 185 cases. The templating adherence was evaluated for stem size and offset option as well as cup size.

**Results:**

Group A showed a lower overall templating adherence with regard to stem size compared to Group B (26.9% vs. 36.2% exact match, *p* = 0.063; 57.5% vs. 71.4% ± 1 size, *p* = 0.007). In female patients templating adherence with regard to stem size was significantly lower in Group A (26.5% vs. 44.4% exact match, *p* = 0.012). For Dorr type B femora, significantly lower templating adherence was observed within Group A with regard to stem size (26.4% vs. 39.6% exact match, *p* = 0.013). No significant differences between both study groups were found with regard to adherence to the templated offset option (60.6% vs. 60.5% exact match, *p* = 0.987) and cup size (43.1% vs. 40.0% exact match, *p* = 0.557).

**Conclusions:**

For both stem types, the overall rate of exactly matching the templated stem sizes was relatively low. However, templating adherence was significantly higher in female patients and in Dorr type B femora with a partially neck-sparing stem, which should be considered by surgeons performing THA using cementless short stems.

**Trial registration:**

This trial was registered at the local ethics committee (Registration Number: 1094/2023).

## Background

Preoperative templating is crucial for the performance of total hip arthroplasty (THA), as it requires preoperative analysis of the patient’s anatomy and biomechanics, allowing anticipation of potentially challenging conditions and increasing overall accuracy of the procedure [1–3]. It also facilitates the selection of an appropriate implant, potentially reducing the risk of implant-related adverse events such as dislocation or leg length discrepancy [4–7]. Furthermore, imprecise stem templating may lead to stem under- or oversizing, which may increase the risk of postoperative stem migration or loosening as well as periprosthetic fractures [8–10].

While 3D-templating techniques are on the rise and seem to offer higher overall accuracy compared to 2D-templating techniques, there are several relevant drawbacks to them such as cost, radiation exposure, duration and overall complexity that leave 2D-templating as a well-established and commonly used templating mode in many arthroplasty centres [7, 11, 12]. In general, there are several factors that might influence templating accuracy such as adequate radiographic positioning, correctly calibrated radiographs or the use of appropriate software [12–15]. In addition, patient-specific characteristics such as body mass index (BMI) or sex appear to influence templating adherence in THA, possibly due to partly sex-specific morphological differences of the proximal femur such as cortical thickness or Dorr type [16–19].

Previous studies have shown reasonable accuracy of preoperative digital 2D-templating in cementless THA when aiming to be at least ± 1 size within the correct stem and cup size [17, 20–22]. However, most of the existing literature on 2D-templating accuracy refers to the use of a straight hip stems [17, 20]. Less data is available on templating adherence using a cementless short stem for THA and few previous studies have investigated the difference in templating adherence between different types of cementless short stems such as neck resecting and partially neck-preserving short stems [12, 16, 23, 24].

As these two stem types feature relevant differences not only in regard to overall design, but also in regard to fixation philosophy and recommended level of femoral neck osteotomy, we aimed to evaluate potential differences in templating adherence between them in primary THA and to assess potential effects of patient-specific characteristics such as BMI, sex and Dorr type as we hypothesized stem design related differences in templating adherence.

## Methods

### Study population

**Fig. 1:**
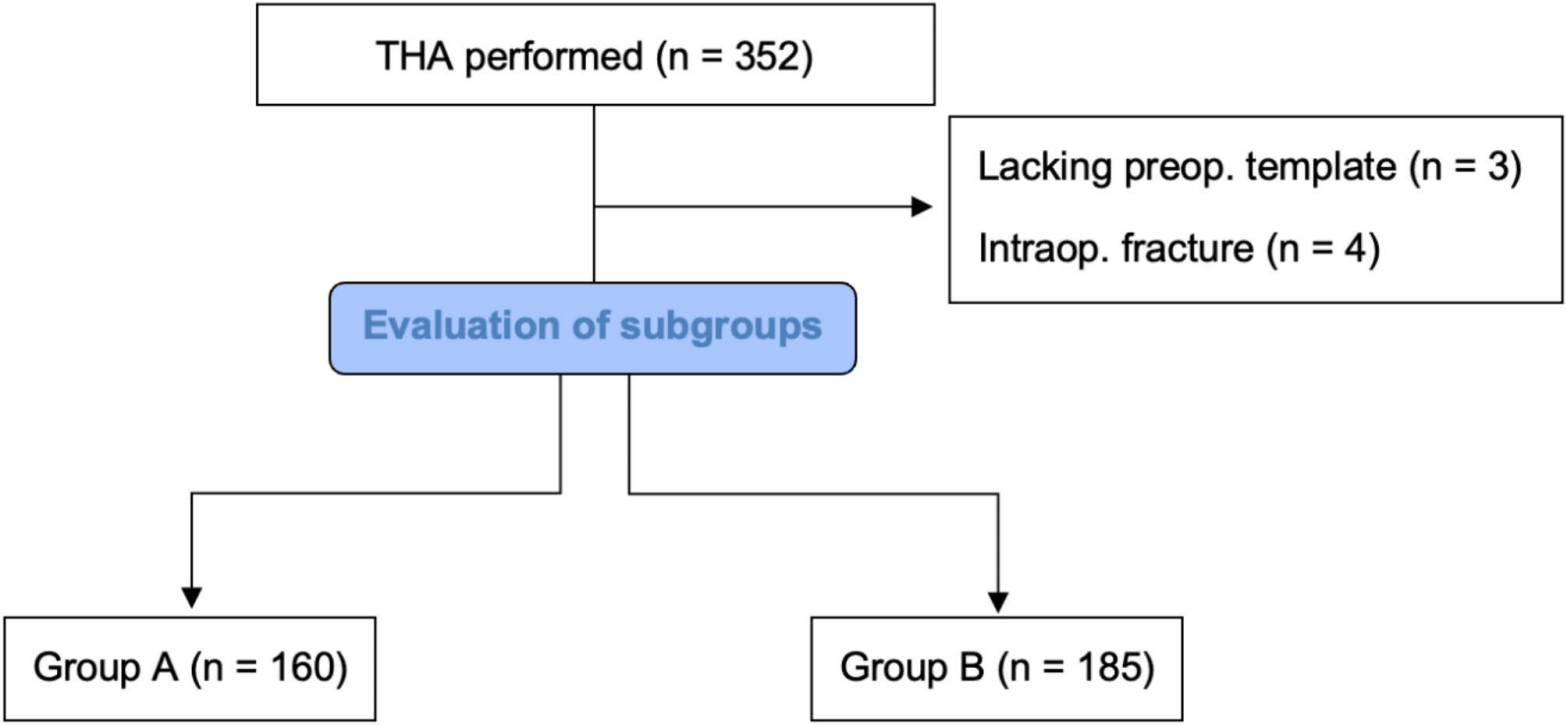
Flowchart regarding formation of the study population Group A is representing patients who received a neck-resecting short stem and Group B is representing patients who received a partially neck-sparing short stem

A consecutive series of 345 primary THAs performed by a single, fellowship-trained surgeon between January 1st 2017 and November 30th 2023 using a minimally invasive direct anterior approach (DAA) to the hip were included in this retrospective cohort study. Each patient’s preoperative radiograph and template as well as the medical records up to hospital discharge were reviewed. Only patients with primary osteoarthritis of the hip, secondary osteoarthritis of the hip following mild hip dysplasia (Crowe ≤ 1) or avascular femoral head necrosis were included in this study. Exclusion criteria were lack of preoperative radiographs or templates, intraoperative adverse events such as fractures, and any other approach to the hip rather than DAA or other implants except the two systems mentioned below (Fig. 1).

Two study groups were retrospectively defined: The Fitmore^®^ hip stem (ZimmerBiomet, Warsaw, IN, USA) in combination with the Allofit^®^/-S press-fit acetabular cup (ZimmerBiomet, Warsaw, IN, USA) was used in 160 cases (Group A) and the ANA.NOVA^®^ Alpha proxy hip stem (ImplanTec GmbH, Moedling, Austria) in combination with the ANA.NOVA^®^ Alpha acetabular cup (ImplanTec GmbH, Moedling, Austria) was used in 185 cases (Group B).

The cementless Fitmore^®^ hip stem can be classified as neck-harming or trochanter sparing short stem due to its recommended level of femoral neck resection [25]. It is made of titanium alloy (TiAl6V4) and features a triple tapered design for press-fit fixation with partial Ti-VPS coating for enhanced osteointegration. It is available in 14 different sizes with 4 different offset options for each size [26].

The cementless ANA.NOVA^®^ Alpha proxy hip stem can be classified as partially neck-sparing short stem due to its recommended level of femoral neck resection [25]. It is made of titanium alloy (TiAl6V4), features a triple tapered trapezoidal design for calcar guided 3-point press-fit fixation with a rough titanium plasma coating and electrochemically applied hydroxyapatite (BONIT^®^) for enhanced osteointegration. It is available in 12 different sizes with 2 different offset options for each size [27].

### Preoperative x-ray technique and templating evaluation

**Fig. 2:**
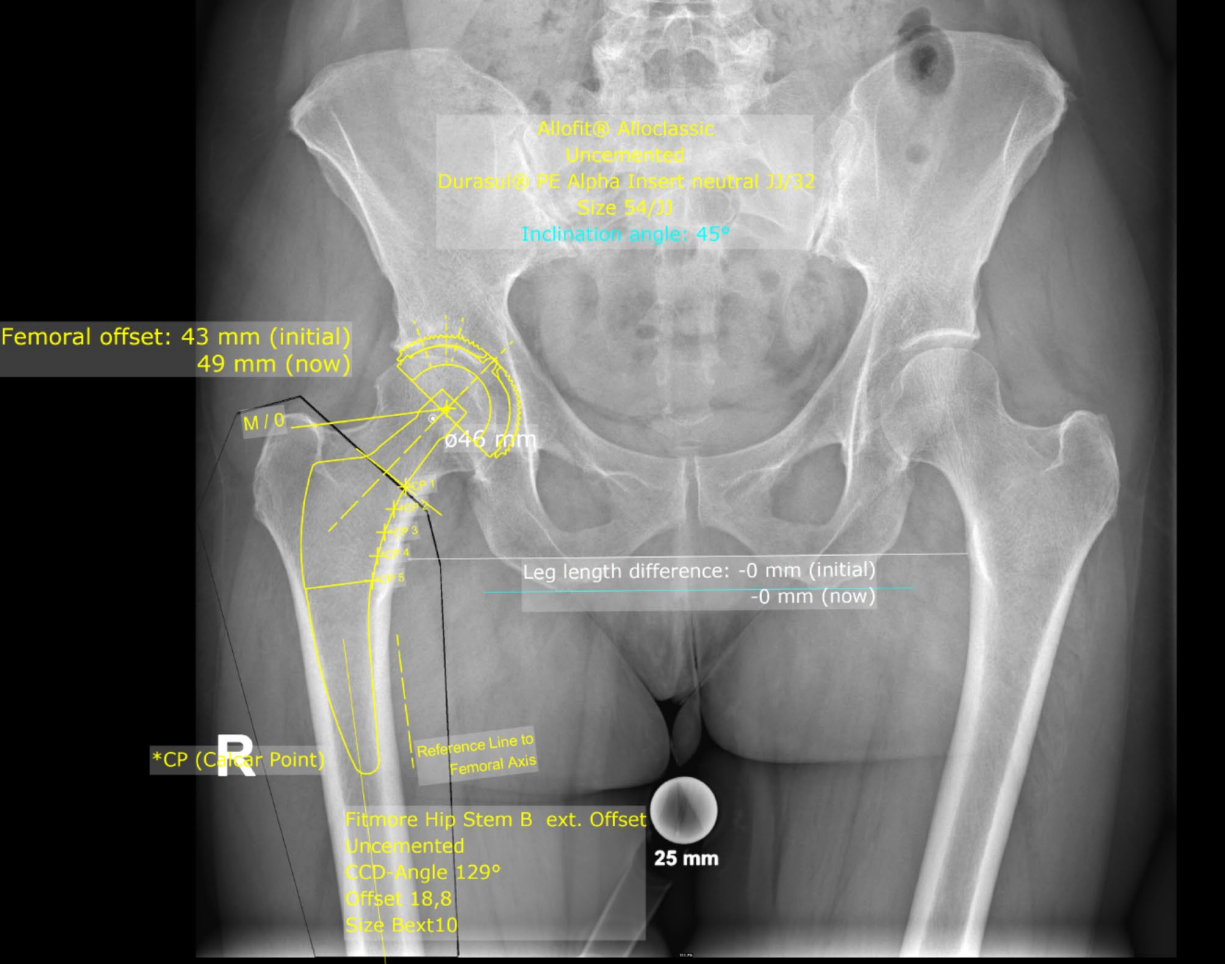
Digital templating of the neck-resecting stem used within Group A

**Fig. 3:**
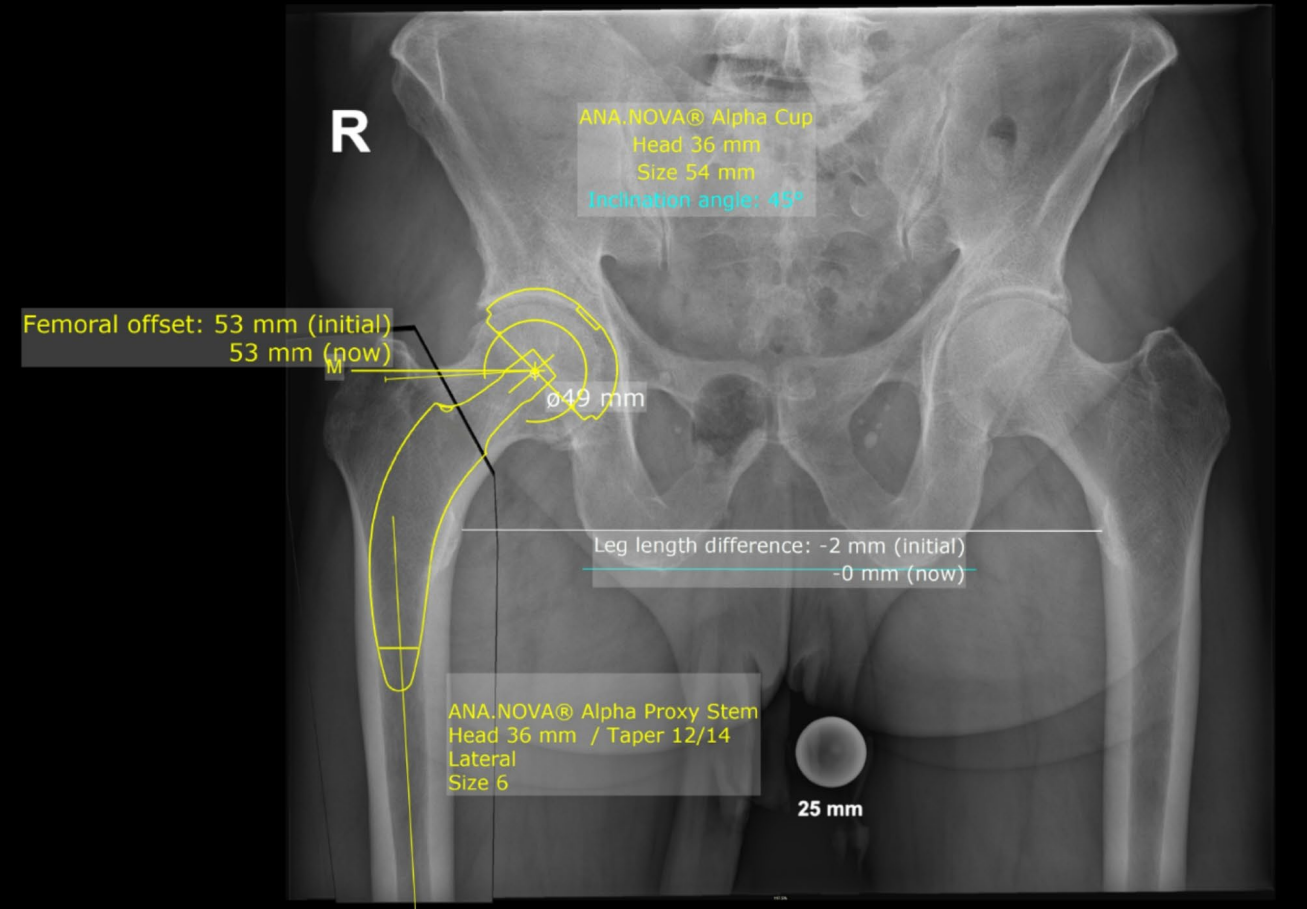
Digital templating of the partially neck preserving stem used within Group B

The preoperative radiographs used for templating were taken with the patient in the standing position, the patient’s legs in 15° internal rotation and the central beam directed at the symphysis pubis with standardized film to focus distance of 1.15 m [20, 28]. A standardized metallic radiopaque ball with a diameter of 25 mm was placed between the patients’ thighs to achieve accurately calibrated radiographs [29]. Preoperative templating aiming for restoration of native hip biomechanics including hip offset and leg length was performed by the surgeon in all investigated cases using MediCAD^®^ Software V5.1 (Hectec GmbH, Germany) [13]. After automatic calibration of the radiograph using the radiopaque ball as reference, the recommended templating workflow was followed by defining the hip’s centre of rotation, the femoral shaft axis as well as the leg length discrepancy. While planning the intended position of the components, the cup was templated at the floor of the acetabulum and the femoral shaft was templated selecting the correct stem size and offset option according to the patient’s anatomy [16]. In case of the Fitmore stem, the aim was to align the stem with the inner cortex of the calcar, with the stem axis aligned with the anatomical axis of the femoral shaft and the stem filling the proximal intramedullary canal (Fig. 2) [16]. In case of the Alpha proxy stem, the aim was to align the stem to the preserved portion of the femoral neck and the medial calcar with additional fixation at the lateral cortex of the femoral shaft. As the Alpha proxy stem has no defined shaft axis, the templated stem position was relative to the planned level of femoral neck resection (Fig. 3). Intraoperative fluoroscopy was used in all cases to avoid stem malpositioning or undersizing. Templating adherence evaluation was performed for stem offset, stem size and cup size. As the Alpha proxy hip stem is available in just two different offset options, the offset templating adherence was evaluated only by checking whether the exact templated offset option was used or not, while stem and cup templating adherence was evaluated by checking whether the used components exactly matched the templated components or if they were within ± 1 or ± 2 sizes, respectively. For study purposes, the Dorr type of each proximal femur was determined from the preoperative radiograph [19].

### Statistical analysis

SPSS version 28 (IBM SPSS statistics, Chicago, IL, USA) was used for performing the statistical analysis. For metric scaled data arithmetic mean value and standard deviation were calculated. Kolmogorov-Smirnov-Test was performed to test for normal distribution.

Chi-Square-Test was performed to analyze categorial parameters while t-Test was performed to analyze normally distributed metric scaled parameters and Man-Whitney-U-Test was conducted to analyze non-normally distributed metric parameters. Univariate binary logistic regression was performed to evaluate the effect of sex, BMI, Dorr type and the planned component size on the templating adherence. A *p* value < 0.05 was considered as statistically significant.

## Results

A total of 345 patients were included in this study. 52.8% of the patients were female and there were no significant differences between the two study groups in terms of basic patient demographics (Table 1).

**Table 1:** Demographics of the study population

	Group A	Group B	*p* value
Number of cases	160	185	-
Sex			0.761
Female	83 (51.9%)	99 (53.5%)	
Male	77 (48.1%)	86 (46.5%)	
Age (years)	66.4 10.8	66.6 11	0.853
Indication			0.567
Primary OA	146 (91.3%)	165 (89.2%)	
AVN	10 (6.3%)	14 (7.6%)	
Hip Dysplasia	4 (2.5%)	4 (2.2%)	
Secondary OA	0 (0.0%)	2 (1.1%)	
Laterality			0.394
Left	80 (50%)	84 (45.4%)	
right	80 (50%)	101 (54.6%)	
BMI	27.6 ± 4.5	27.2 ± 4.7	0.456

Overall, templating adherence for femoral stem size was tendentially lower in Group A compared to Group B (26.9% vs. 36.2%; *p* = 0.063) with significantly lower templating adherence within ± 1 (57.5% vs. 71.4%; *p* = 0.007) and ± 2 stem sizes (76.9% vs. 94.1%; *p* < 0.001). Similar templating adherence was found for the correct offset option and cup size without significant differences between the two study groups.

Comparing templating adherence in female and male patients between the two study groups, the templating adherence for the femoral stem size was significantly lower in females in Group A compared to females in Group B for exact stem size (26.5% vs. 44.4%, *p* = 0.012), ± 1 size (57.8% vs. 78.8%, *p* = 0.002) and ± 2 sizes (75.9% vs. 96.0%, *p* < 0.001), while no significant differences were found for the offset option and cup size as well as for male patients in general, except for stem size within ± 2 sizes (77.9% vs. 91.9%, *p* = 0.012; Table 2).

**Table 2:** Comparison of the templating adherence between female and male patients of the two study groups

	Group A (*n* = 160)	Group B (*n* = 185)	*p* Value	Females Group A (*n* = 83)	Females Group B (*n* = 99)	*p* Value	Males Group A (*n* = 77)	Males Group B (*n* = 86)	*p* Value
Offset option									
Exact match	97 (60.6%)	112 (60.5%)	0.987	55 (66.3%)	60 (60.6%)	0.430	42 (54.5%)	52 (60.4%)	0.445
Stem size									
Exact match	43 (26.9%)	67 (36.2%)	0.063	22 (26.5%)	44 (44.4%)	**0.012**	21 (27.3%)	23 (26.7%)	0.940
± 1 size	92 (57.5%)	132 (71.4%)	**0.007**	48 (57.8%)	78 (78.8%)	**0.002**	44 (57.1%)	54 (62.8%)	0.462
± 2 sizes	123 (76.9%)	174 (94.1%)	**< 0.001**	63 (75.9%)	95 (96.0%)	**< 0.001**	60 (77.9%)	79 (91.9%)	**0.012**
Cup size									
Exact match	69 (43.1%)	74 (40.0%)	0.557	36 (43.4%)	45 (45.5%)	0.778	33 (42.9%)	29 (33.7%)	0.230
± 1 size	122 (76.3%)	149 (80.5%)	0.333	65 (78.3%)	84 (84.8%)	0.254	57 (74.0%)	65 (75.6%)	0.819
± 2 sizes	154 (96.3%)	177 (95.7%)	0.787	80 (96.4%)	95 (96.0%)	0.882	74 (96.1%)	82 (95.3%)	0.812

When comparing templating adherence in female and male patients within the two study groups, no significant sex related differences were found in Group A, whereas in Group B femoral stem size templating adherence was significantly lower in male patients (Table 3).

**Table 3:** Comparison of the templating adherence between female and male patients within the two study groups

	Females (*n* = 182)	Males (*n* = 163)	*p* Value	Females Group A (*n* = 83)	Males Group A (*n* = 77)	*p* Value	Females Group B (*n* = 99)	Males Group B (*n* = 86)	*p* Value
Offset option									
Exact match	115 (63.2%)	94 (57.7%)	0.295	55 (66.3%)	42 (54.5%)	0.147	60 (60.1%)	52 (60.5%)	1.000
Stem size									
Exact match	66 (36.3%)	44 (27.0%)	0.065	22 (26.5%)	21 (27.3%)	1.000	44 (44.4%)	23 (26.7%)	**0.014**
± 1 size	126 (69.2%)	98 (69.1%)	0.077	48 (57.8%)	44 (57.1%)	1.000	78 (78.8%)	54 (62.8%)	**0.022**
± 2 sizes	158 (86.8%)	139 (85.3%)	0.680	63 (75.9%)	60 (77.9%)	0.852	95 (96.0%)	86 (100.0%)	0.351
Cup size									
Exact match	81 (44.5%)	62 (38.0%)	0.223	36 (43.4%)	33 (42.9%)	1.000	45 (45.5%)	29 (33.7%)	0.132
± 1 size	149 (81.9%)	122 (74.8%)	0.113	65 (78.3%)	57 (74.0%)	0.579	84 (84.8%)	65 (75.6%)	0.137
± 2 sizes	175 (96.2%)	156 (95.7%)	0.833	80 (96.4%)	74 (96.1%)	1.000	95 (96.0%)	82 (95.3%)	1.000

Evaluation of the templating adherence in relation to the Dorr classification showed a significantly lower templating adherence for Dorr type B femora within Group A for exact stem size (26.4% vs. 39.6%, *p* = 0.013), ± 1 size (57.4% vs. 73.2%, *p* = 0.003) and ± 2 sizes (76.4% vs. 94.5%, *p* < 0.001; Table 4).

**Table 4:** Comparison of the templating adherence of the stem based on the Dorr types

	Dorr A			Dorr B			Dorr C		
	Group A (*n* = 10)	Group B (*n* = 17)	*p* Value	Group A (*n* = 148)	Group B (*n* = 164)	*p* Value	Group B (*n* = 2)	Group B (*n* = 4)	*p* Value
Stem size									
Exact match	2 (20.0%)	2 (11.8%)	0.561	39 (26.4%)	65 (39.6%)	**0.013**	2 (100.0%)	0 (0.0%)	**0.014**
± 1 size	5 (50.0%)	11 (64.7%)	0.453	85 (57.4%)	120 (73.2%)	**0.003**	2 (100.0%)	1 (25.0%)	0.083
± 2 sizes	8 (80.0%)	16 (94.1%)	0.260	113 (76.4%)	155 (94.5%)	**< 0.001**	2 (100.0%)	3 (75.0%)	0.439

The logistic regression showed a significant influence of sex (OR: 2.2, 95% CI: 1.2–4.1; *p* = 0.013), BMI (OR: 0.90, 95% CI: 0.8–1.0; *p* = 0.005) and Dorr type (OR: 6.237, 95% CO: 1.405–27.682; *p* = 0.016) in regard to exactly matching the templated stem size within Group B, but not within Group A (Table 5).

**Table 5:** Results of the regression analysis including the Odds-Ratio (OR) and the 95%-Confidence-Interval (95%-CI)

	OR overall (95%-CI)	*p* Value	OR Group A (95%-CI)	*p* Value	OR Group B (95%-CI)	*p* Value
Offset exact match						
Sex female	1.260 (0.817–1942)	0.295	1.637 (0.864–3.101)	0.131	1.006 (0.557–1.817)	0.984
BMI	1.009 (0.962–1.058)	0.705	0.985 (0.918–1.057)	0.682	1.029 (0.965–1.098)	0.381
Stem exact match						
Sex female	1.539 (0.972–2.436)	0.066	0.913 (0.478–1.935)	0.913	2.191 (1.178–4.076)	**0.013**
BMI	0.918 (0.869–0.969)	**0.002**	0.945 (0.870–1.027)	0.185	0.901 (0.837–0.969)	**0.005**
Stem size	0.947 (0.853–1.051)	0.304	1.016 (0.867–1.191)	0.842	0.931 (0.801–1.083)	0.354
Dorr type B	2.250 (0.901–5.620)	0.083	0.716 (0.204–2.509)	0.601	6.237 (1.405–27.682)	**0.016**
Cup exact match						
Sex female	0.765 (0.498–1.177)	0.224	0.979 (0.523–1.832)	0.947	0.611 (0.336–1.109)	0.105
BMI	1.034 (0.986–1.085)	0.171	1.018 (0.949–1.092)	0.617	1.049 (0.982–1.120)	0.154
Cup size	1.043 (0.972–1.118)	0.240	1.007 (0.908–1.116)	0.895	1.086 (0.983–1.199)	0.103

## Discussion

The main findings of the current study are a higher overall templating accuracy with respect to stem size for a partially neck-sparing stem compared to a neck-resecting short stem with significant differences especially within female patients and in Dorr type B femora, in whom a significantly higher templating adherence was found when performing THA using a partially neck-preserving short stem. No significant differences in templating adherence were found for stem offset option and cup size within this study.

This study revealed an overall templating adherence rate of 26.9% in the neck-resecting group and 36.2% in the partially neck-sparing group for exactly matching the templated stem size, which is comparable to the results of other studies investigating the accuracy of preoperative 2D templating [13, 24]. However, there are also studies investigating cementless short stems that report higher 2D templating adherence [30]. When comparing the templating adherence found within this study to studies investigating the templating accuracy of straight stems, the templating accuracy for the two types of short stems investigated is notably lower as other authors report templating accuracies of 52–78% for cementless straight stems with diaphyseal anchorage [20, 31, 32]. Although it could be assumed that conventional straight stems with diaphyseal anchorage are easier to template and therefore more accurate in terms of templating adherence compared to short stems with combined meta-diaphyseal anchorage, there are also reports claiming no difference in planning accuracy between these two types of stems [33].

In this study, a higher BMI was associated with significantly poorer templating adherence, as was male sex in patients receiving a partially neck-sparing short stem. While the use of a scaling marker including correct placement and calibration appears to be an essential foundation for achieving accurate preoperative templates [34, 35], there are several other factors that influence the accuracy of preoperative digital templating in THA. For example, according to previous studies, obesity appears to negatively influence 2D-digital templating accuracy of the femoral stem but not necessarily the templating accuracy of the acetabular cup, which was also found within the present study [12, 17]. In addition, gender appears to potentially influence femoral stem size templating adherence, as other studies have found a higher templating accuracy regarding the femoral stem size for female patients with no significant differences regarding the acetabular cup size, which is also consistent with the results of the present study [20]. Whilst sex and BMI may influence the magnification factor of the radiograph and therefore potentially affect the accuracy of the calibration device, which may be a possible explanation for the findings of the present study, there are also studies that have found no significant influence of sex or BMI on the digital templating accuracy [36–38].

According to the results of this study, a partially neck-preserving short stem seems to provide a higher templating accuracy, especially in female patients, whereas no significant differences in templating adherence for stem size were found between the two investigated stem types within male patients with the exception of matching the preoperatively templated stem size within 2 sizes. Due to the study design, we can’t provide a definitive causal explanation for this finding. While sex specific differences in the proximal femur and soft tissues surrounding the hip could potentially influence intraoperative stem positioning and therefore indirectly templating, no conclusions in that regard can be drawn from the results of this study [39, 40]. Within this study, templating adherence was significantly higher in Dorr type B femora when using a partially neck-preserving stem for THA, which contrasts with the findings of Mevorach et al., who found no significant effect of the Dorr classification on templating adherence [41]. Finally, one factor to consider is the high variability of possible different stem positions when using a cementless short stem, which could also potentially influence the templating accuracy of the femoral stem [42]. Interestingly, no significant sex or implant specific differences with regard to templating accuracy of the stem’s offset option were found in this study, despite the neck-resecting stem used within this study is available in four different offset options, while the partially neck-sparing stem used within this study is available in two offset options, which is in contrast to other reports that found significant sex related differences regarding the templating adherence of the stem’s offset option [16].

There are a number of important limitations to this study that need to be considered when interpreting the results. The retrospective study design and the lack of specific data regarding the individual anatomical conditions of the pelvis and the proximal femur, other than Dorr type assessment limit the value of this study as the findings can only be described and discussed but not definitively and causally explained. In addition, the majority of the patients within this study population had Dorr type B femora, which limits the findings of this study and potential conclusions with regard to Dorr type A and C femora. The lack of a postoperative follow-up represents another limitation of the present study. Furthermore, only a single fellowship-trained surgeon, who performed not only the templating but also THA in all investigated cases, was evaluated within this study, which potentially leaves room for individual errors in templating technique and therefore limits the applicability of this study’s findings to other surgeons and orthopedic centers. No control group using a traditional straight stem was evaluated in this study, which represents another limitation. Finally, the neck-resecting stem used in this study is available in 14 sizes, while the partially neck-sparing stem used within this study is only available in 12 sizes, which doesn’t influence the sex specific differences regarding the accuracy of the templated femoral stem size found within the partially neck-sparing group, but may influence the differences in the accuracy of the templated femoral stem size between both study groups. In addition, as mentioned above, the neck-resecting stem used in this study is available in four different offset options, whereas the partially neck-sparing stem used in this study is only available in two different offset options, which– despite the lack of significant differences regarding templated offset adherence– may influence the results of this study.

## Conclusions

Overall, digital 2-D templating of a neck-resecting short stem shows a relatively low accuracy in exactly matching the actual femoral stem size. For a partially neck-sparing short stem, exact digital templating appears to be challenging especially in male patients and in femora other than Dorr type B. In addition, a high BMI appears to negatively affect templating accuracy for both studied types of cementless short stems. Surgeons performing THA with cementless short stems should be aware of these factors that may affect digital templating accuracy.

## Data Availability

The dataset used and analysed during the current study is available from the corresponding author on reasonable request.

## Abbreviations

THA: Total hip arthroplasty
BMI: Body Mass Index
